# 4-Acetamido-3-nitro­phenyl acetate

**DOI:** 10.1107/S1600536809015797

**Published:** 2009-05-07

**Authors:** Zhun Gu, Wei Cheng

**Affiliations:** a Department of Chemical Engineering, Chien-shiung Institute of Technology, Suzhou 215411, People’s Republic of China; b Department of Applied Chemistry, College of Science, Nanjing University of Technology, Nanjing 210009, People’s Republic of China

## Abstract

In the mol­ecule of the title compound, C_10_H_10_N_2_O_5_, intra­molecular C—H⋯O inter­actions result in the formation of a five- and a six-membered ring. The five-membered ring is planar and is oriented at a dihedral angle of 0.34 (3)° with respect to the plane of the aromatic ring, while the six-membered ring has a twist conformation. In the crystal structure, inter­molecular C—H⋯O inter­actions link the mol­ecules into chains.

## Related literature

For a related structure, see: Gu (2007 ▶). For bond-length data, see: Allen *et al.* (1987 ▶).
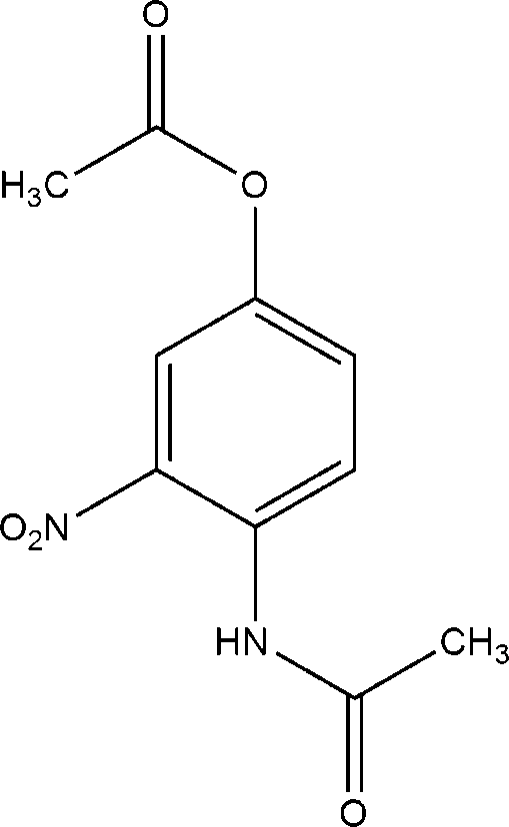


## Experimental

### 

#### Crystal data

C_10_H_10_N_2_O_5_
*M* *_r_* = 238.20
Monoclinic, 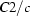

*a* = 24.859 (5) Å
*b* = 4.7060 (9) Å
*c* = 19.773 (4) Å
β = 108.67 (3)°
*V* = 2191.4 (8) Å^3^
*Z* = 8
Mo *K*α radiation
μ = 0.12 mm^−1^
*T* = 298 K
0.30 × 0.10 × 0.10 mm

#### Data collection

Enraf–Nonius CAD-4 diffractometer
Absorption correction: ψ scan (North *et al.*, 1968 ▶) *T*_min_ = 0.966, *T*_max_ = 0.988
2039 measured reflections
1992 independent reflections
1310 reflections with *I* > 2σ(*I*)
*R*_int_ = 0.021
3 standard reflections frequency: 120 min intensity decay: 1%

#### Refinement

*R*[*F* ^2^ > 2σ(*F* ^2^)] = 0.064
*wR*(*F* ^2^) = 0.187
*S* = 1.00
1992 reflections
136 parameters
H-atom parameters constrained
Δρ_max_ = 0.51 e Å^−3^
Δρ_min_ = −0.56 e Å^−3^

### 

Data collection: *CAD-4 Software* (Enraf–Nonius, 1985 ▶); cell refinement: *CAD-4 Software*; data reduction: *XCAD4* (Harms & Wocadlo, 1995 ▶); program(s) used to solve structure: *SHELXS97* (Sheldrick, 2008 ▶); program(s) used to refine structure: *SHELXL97* (Sheldrick, 2008 ▶); molecular graphics: *ORTEP-3 for Windows* (Farrugia, 1997 ▶); software used to prepare material for publication: *SHELXL97*.

## Supplementary Material

Crystal structure: contains datablocks I, global. DOI: 10.1107/S1600536809015797/hk2677sup1.cif

Structure factors: contains datablocks I. DOI: 10.1107/S1600536809015797/hk2677Isup2.hkl

Additional supplementary materials:  crystallographic information; 3D view; checkCIF report

## Figures and Tables

**Table 1:** Hydrogen-bond geometry (Å, °)

*D*—H⋯*A*	*D*—H	H⋯*A*	*D*⋯*A*	*D*—H⋯*A*
C4—H4*A*⋯O2	0.93	2.33	2.647 (4)	100
C7—H7*A*⋯O5	0.93	2.35	2.836 (4)	113
C10—H10*C*⋯O5^i^	0.96	2.59	3.300 (4)	130

Symmetry code: (i) 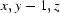.

**Fig. 1.**
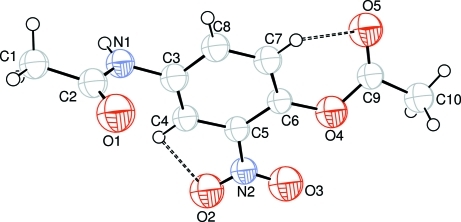
The molecular structure of the title molecule, with the atom-numbering scheme. Hydrogen bonds are shown as dashed lines.

## supplementary crystallographic information

### Comment

The title compound is an important medical intermediate used to synthesize 3,4-diaminophenol, which is the main raw material of luxabendazole (Gu, 2007). We report herein the crystal structure of the title compound, which is of interest to us in the field.

In the molecule of the title compound (Fig 1), the bond lengths (Allen *et al.*,  1987) and angles are within normal ranges. Ring A (C3-C8) is, of course, planar. Intramolecular C-H···O interactions (Table 1) result in the formations of five- and six-membered rings: B (O2/N2/C4/C5/H4A) and C (O4/O5/C5-C7/C9/H7A). Ring B is planar and it is oriented with respect to ring A at a dihedral angle of 0.34 (3)°, while ring C has a twisted conformation.

In the crystal structure, intermolecular C-H···O interactions (Table 1) link the molecules into chains, in which they may be effective in the stabilization of the structure.

### Experimental

The title compound was prepared by the reaction of 4-aminophenol, fuming nitric acid and acetic anhydride (Gu, 2007). Crystals suitable for X-ray analysis were obtained by dissolving the title compound (0.2 g) in ethanol (25 ml) and evaporating the solvent slowly at room temperature for about 2 d.

### Refinement

H atoms were positioned geometrically, with N-H = 0.86 Å (for NH) and C-H = 0.93 and 0.96 Å for aromatic and methyl H, respectively, and constrained to ride on their parent atoms, with U_iso_(H) = xU_eq_(C,N), where x = 1.5 for methyl H and x = 1.2 for all other H atoms.

### Crystal data

**Table:**

C_10_H_10_N_2_O_5_	*F*(000) = 992
*M**_r_* = 238.20	*D*_x_ = 1.444 Mg m^−^^3^
Monoclinic, *C*2/*c*	Mo *K*α radiation, λ = 0.71073 Å
Hall symbol: -C 2yc	Cell parameters from 25 reflections
*a* = 24.859 (5) Å	θ = 9–13°
*b* = 4.7060 (9) Å	µ = 0.12 mm^−^^1^
*c* = 19.773 (4) Å	*T* = 298 K
β = 108.67 (3)°	Needle, colorless
*V* = 2191.4 (8) Å^3^	0.30 × 0.10 × 0.10 mm
*Z* = 8	

### Data collection

**Table:**

Enraf–Nonius CAD-4 diffractometer	1310 reflections with *I* > 2σ(*I*)
Radiation source: fine-focus sealed tube	*R*_int_ = 0.021
graphite	θ_max_ = 25.3°, θ_min_ = 1.7°
ω/2θ scans	*h* = 0→29
Absorption correction: ψ scan (North *et al.*, 1968)	*k* = 0→5
*T*_min_ = 0.966, *T*_max_ = 0.988	*l* = −23→22
2039 measured reflections	3 standard reflections every 120 min
1992 independent reflections	intensity decay: 1%

### Refinement

**Table:**

Refinement on *F*^2^	Secondary atom site location: difference Fourier map
Least-squares matrix: full	Hydrogen site location: inferred from neighbouring sites
*R*[*F*^2^ > 2σ(*F*^2^)] = 0.064	H-atom parameters constrained
*wR*(*F*^2^) = 0.187	*w* = 1/[σ^2^(*F*_o_^2^) + (0.1*P*)^2^ + 1.4*P*] where *P* = (*F*_o_^2^ + 2*F*_c_^2^)/3
*S* = 1.00	(Δ/σ)_max_ < 0.001
1992 reflections	Δρ_max_ = 0.51 e Å^−^^3^
136 parameters	Δρ_min_ = −0.56 e Å^−^^3^
0 restraints	Extinction correction: *SHELXL97* (Sheldrick, 2008)
Primary atom site location: structure-invariant direct methods	Extinction coefficient: 0.091 (8)

### Special details

**Table:**

Geometry. All e.s.d.'s (except the e.s.d. in the dihedral angle between two l.s. planes) are estimated using the full covariance matrix. The cell e.s.d.'s are taken into account individually in the estimation of e.s.d.'s in distances, angles and torsion angles; correlations between e.s.d.'s in cell parameters are only used when they are defined by crystal symmetry. An approximate (isotropic) treatment of cell e.s.d.'s is used for estimating e.s.d.'s involving l.s. planes.
Refinement. Refinement of *F*^2^ against ALL reflections. The weighted *R*-factor *wR* and goodness of fit *S* are based on *F*^2^, conventional *R*-factors *R* are based on *F*, with *F* set to zero for negative *F*^2^. The threshold expression of *F*^2^ > σ(*F*^2^) is used only for calculating *R*-factors(gt) *etc*. and is not relevant to the choice of reflections for refinement. *R*-factors based on *F*^2^ are statistically about twice as large as those based on *F*, and *R*- factors based on ALL data will be even larger.

### Fractional atomic coordinates and isotropic or equivalent isotropic displacement parameters (Å^2^)

**Table:**

	*x*	*y*	*z*	*U*_iso_*/*U*_eq_	
N1	0.07647 (10)	0.5724 (5)	0.10146 (12)	0.0515 (4)	
H1A	0.0516	0.7061	0.0897	0.062*	
N2	0.06554 (11)	−0.0326 (5)	−0.09742 (14)	0.0514 (7)	
O1	0.12640 (12)	0.2734 (6)	0.18301 (13)	0.0844 (9)	
O2	0.02074 (10)	−0.1112 (6)	−0.09213 (14)	0.0857 (9)	
O3	0.08434 (11)	−0.1300 (6)	−0.14164 (15)	0.0872 (9)	
O4	0.17417 (10)	0.2065 (5)	−0.10210 (12)	0.0688 (7)	
O5	0.21944 (11)	0.6153 (5)	−0.11162 (14)	0.0770 (8)	
C1	0.06037 (13)	0.5863 (7)	0.21107 (15)	0.0515 (4)	
H1B	0.0723	0.5004	0.2576	0.077*	
H1C	0.0204	0.5560	0.1888	0.077*	
H1D	0.0680	0.7866	0.2156	0.077*	
C2	0.09173 (14)	0.4575 (7)	0.16704 (16)	0.0515 (4)	
C3	0.10194 (13)	0.4694 (7)	0.05257 (16)	0.0515 (4)	
C4	0.07426 (12)	0.2714 (6)	0.00398 (15)	0.0457 (7)	
H4A	0.0399	0.1961	0.0048	0.055*	
C5	0.09830 (12)	0.1834 (6)	−0.04697 (15)	0.0441 (7)	
C6	0.14971 (11)	0.2930 (6)	−0.05065 (15)	0.0427 (7)	
C7	0.17661 (12)	0.4918 (6)	0.00190 (17)	0.0512 (8)	
H7A	0.2116	0.5645	0.0029	0.061*	
C8	0.15321 (13)	0.5825 (6)	0.05171 (16)	0.0514 (8)	
H8A	0.1716	0.7196	0.0850	0.062*	
C9	0.20627 (12)	0.3744 (6)	−0.13076 (16)	0.0484 (7)	
C10	0.22342 (15)	0.2336 (7)	−0.18863 (18)	0.0607 (9)	
H10A	0.2458	0.3629	−0.2061	0.091*	
H10B	0.1901	0.1808	−0.2270	0.091*	
H10C	0.2454	0.0667	−0.1699	0.091*	

### Atomic displacement parameters (Å^2^)

**Table:**

	*U*^11^	*U*^22^	*U*^33^	*U*^12^	*U*^13^	*U*^23^
N1	0.0594 (9)	0.0497 (9)	0.0486 (8)	0.0021 (7)	0.0217 (7)	0.0021 (7)
N2	0.0494 (14)	0.0471 (15)	0.0587 (16)	−0.0106 (12)	0.0185 (12)	−0.0027 (13)
O1	0.0895 (18)	0.093 (2)	0.0743 (17)	0.0254 (16)	0.0320 (14)	0.0246 (15)
O2	0.0725 (16)	0.093 (2)	0.100 (2)	−0.0430 (15)	0.0402 (15)	−0.0344 (16)
O3	0.0887 (18)	0.095 (2)	0.0935 (19)	−0.0414 (16)	0.0506 (16)	−0.0460 (16)
O4	0.0795 (16)	0.0577 (14)	0.0814 (17)	−0.0073 (12)	0.0426 (14)	−0.0024 (12)
O5	0.109 (2)	0.0442 (14)	0.0964 (19)	−0.0279 (13)	0.0595 (16)	−0.0069 (13)
C1	0.0594 (9)	0.0497 (9)	0.0486 (8)	0.0021 (7)	0.0217 (7)	0.0021 (7)
C2	0.0594 (9)	0.0497 (9)	0.0486 (8)	0.0021 (7)	0.0217 (7)	0.0021 (7)
C3	0.0594 (9)	0.0497 (9)	0.0486 (8)	0.0021 (7)	0.0217 (7)	0.0021 (7)
C4	0.0444 (15)	0.0400 (16)	0.0561 (17)	−0.0079 (13)	0.0207 (13)	−0.0007 (14)
C5	0.0483 (15)	0.0337 (14)	0.0490 (16)	−0.0059 (13)	0.0139 (13)	−0.0002 (13)
C6	0.0463 (15)	0.0332 (14)	0.0508 (16)	−0.0008 (13)	0.0184 (13)	0.0066 (13)
C7	0.0479 (16)	0.0421 (16)	0.0637 (19)	−0.0107 (13)	0.0181 (15)	−0.0017 (15)
C8	0.0601 (18)	0.0422 (17)	0.0498 (17)	−0.0059 (15)	0.0146 (14)	−0.0020 (14)
C9	0.0521 (17)	0.0392 (17)	0.0585 (18)	−0.0001 (14)	0.0239 (14)	0.0058 (14)
C10	0.076 (2)	0.0516 (19)	0.068 (2)	−0.0007 (17)	0.0421 (18)	0.0031 (17)

### Geometric parameters (Å, °)

**Table:**

O1—C2	1.191 (4)	C3—C4	1.357 (4)
O4—C9	1.368 (3)	C3—C8	1.386 (4)
O4—C6	1.402 (3)	C4—C5	1.389 (4)
O5—C9	1.207 (3)	C4—H4A	0.9300
N1—C2	1.343 (4)	C5—C6	1.402 (4)
N1—C3	1.401 (4)	C6—C7	1.399 (4)
N1—H1A	0.8600	C7—C8	1.363 (4)
N2—O3	1.206 (3)	C7—H7A	0.9300
N2—O2	1.209 (3)	C8—H8A	0.9300
N2—C5	1.474 (4)	C9—C10	1.497 (4)
C1—C2	1.473 (4)	C10—H10A	0.9600
C1—H1B	0.9600	C10—H10B	0.9600
C1—H1C	0.9600	C10—H10C	0.9600
C1—H1D	0.9600		
			
C9—O4—C6	125.4 (2)	C4—C5—C6	122.6 (3)
C2—N1—C3	118.5 (3)	C4—C5—N2	115.1 (2)
C2—N1—H1A	120.8	C6—C5—N2	122.3 (3)
C3—N1—H1A	120.8	C7—C6—C5	115.7 (3)
O2—N2—C5	118.5 (3)	C7—C6—O4	121.2 (2)
O3—N2—O2	121.8 (3)	C5—C6—O4	123.0 (3)
O3—N2—C5	119.6 (2)	C8—C7—C6	122.2 (3)
C2—C1—H1B	109.5	C8—C7—H7A	118.9
C2—C1—H1C	109.5	C6—C7—H7A	118.9
H1B—C1—H1C	109.5	C7—C8—C3	119.7 (3)
C2—C1—H1D	109.5	C7—C8—H8A	120.2
H1B—C1—H1D	109.5	C3—C8—H8A	120.2
H1C—C1—H1D	109.5	O5—C9—O4	123.2 (3)
O1—C2—N1	120.4 (3)	O5—C9—C10	122.7 (3)
O1—C2—C1	128.2 (3)	O4—C9—C10	114.1 (3)
N1—C2—C1	111.5 (3)	C9—C10—H10A	109.5
C4—C3—C8	121.0 (3)	C9—C10—H10B	109.5
C4—C3—N1	119.2 (3)	H10A—C10—H10B	109.5
C8—C3—N1	119.7 (3)	C9—C10—H10C	109.5
C3—C4—C5	118.7 (3)	H10A—C10—H10C	109.5
C3—C4—H4A	120.7	H10B—C10—H10C	109.5
C5—C4—H4A	120.7		
			
C3—N1—C2—O1	0.4 (5)	N2—C5—C6—C7	−178.6 (3)
C3—N1—C2—C1	−179.1 (3)	C4—C5—C6—O4	−179.7 (3)
C2—N1—C3—C4	97.3 (4)	N2—C5—C6—O4	−0.2 (4)
C2—N1—C3—C8	−86.5 (4)	C9—O4—C6—C7	−33.5 (4)
C8—C3—C4—C5	0.0 (5)	C9—O4—C6—C5	148.2 (3)
N1—C3—C4—C5	176.2 (3)	C5—C6—C7—C8	−2.7 (4)
C3—C4—C5—C6	−0.7 (5)	O4—C6—C7—C8	178.9 (3)
C3—C4—C5—N2	179.8 (3)	C6—C7—C8—C3	2.2 (5)
O3—N2—C5—C4	−177.0 (3)	C4—C3—C8—C7	−0.7 (5)
O2—N2—C5—C4	1.6 (4)	N1—C3—C8—C7	−176.9 (3)
O3—N2—C5—C6	3.5 (4)	C6—O4—C9—O5	3.9 (5)
O2—N2—C5—C6	−178.0 (3)	C6—O4—C9—C10	−175.7 (3)
C4—C5—C6—C7	1.9 (4)		

### Hydrogen-bond geometry (Å, °)

**Table:**

*D*—H···*A*	*D*—H	H···*A*	*D*···*A*	*D*—H···*A*
C4—H4A···O2	0.93	2.33	2.647 (4)	100
C7—H7A···O5	0.93	2.35	2.836 (4)	113
C10—H10C···O5^i^	0.96	2.59	3.300 (4)	130

Symmetry codes: (i) *x*, *y*−1, *z*.
